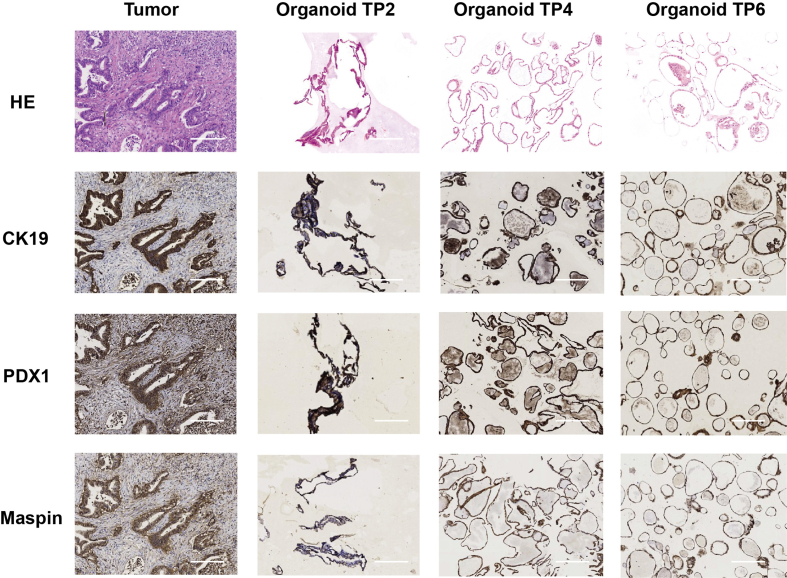# PRKRA promotes pancreatic cancer progression by upregulating MMP1 transcription via the NF-κB pathway

**DOI:** 10.1016/j.heliyon.2023.e17194

**Published:** 2023-06-10

**Authors:** Jiangdong Qiu, Mengyu Feng, Gang Yang, Dan Su, Fangyu Zhao, Yueze Liu, Jinxin Tao, Wenhao Luo, Taiping Zhang

**Affiliations:** a Department of General Surgery, State Key Laboratory of Complex Severe and Rare Diseases, Peking Union Medical College Hospital, Chinese Academy of Medical Sciences and Peking Union Medical College, Beijing, 100730, China; b Key Laboratory of Research in Pancreatic Tumor, Chinese Academy of Medical Sciences, Beijing, 100730, China; c Gastrointestinal Cancer Center, Key Laboratory of Carcinogenesis and Translational Research (Ministry of Education), Peking University Cancer Hospital and Institute, Beijing, 100142, China

**Keywords:** PRKRA, Pancreatic cancer, Organoids, Progression, Biomarker

## Abstract

**Objective:**

Pancreatic cancer (PC) is highly malignant, but the underlying mechanisms of cancer progression remain unclear. PRKRA is involved in cellular stress response, but its role in PC was unknown.

**Methods:**

The expression of PRKRA between normal and tumor tissues were compared, and the prognostic value of PRKRA was evaluated. SiRNA and plasmids were applied to investigate the effects of PRKRA on PC cells. Organoids and cell lines with knockout and overexpression of PRKRA were established by CRISPR/Cas9 and lentivirus. The effects of PRKRA on PC were evaluated in vivo by cell-derived xenografts. The downstream genes of PRKRA were screened by transcriptome sequencing. The regulation of the target gene was validated by RT-qPCR, western blot, ChIP and dual luciferase reporter assay. Besides, the correlation between PRKRA and gemcitabine sensitivity was investigated by PC organoids.

**Results:**

PRKRA was significantly overexpressed in PC tissues and independently associated with poor prognosis. PRKRA promoted the proliferation, migration, and chemoresistance of PC cells. The proliferation of PC organoids was decreased by PRKRA knockout. The growth and chemoresistance of xenografts were increased by PRKRA overexpression. Mechanistically, PRKRA upregulated the transcription of MMP1 via NF-κB pathway. ChIP and dual luciferase reporter assay showed that NF-κB subunit P65 could bind to the promoter of MMP1. The sensitivity of PC organoids to gemcitabine was negatively correlated with the expression of PRKRA and MMP1.

**Conclusions:**

Our study indicated that the PRKRA/NF-κB/MMP1 axis promoted the progression of PC and may serve as a potential therapeutic target and prognosis marker.

## 1 Introduction

As one of the most lethal malignancies, the 5-year survival rate of PC is only 12% and ranks as the fourth leading cause of cancer-related death [1]. Although cancer treatment has achieved great progress for decades, PC is still resistant to multiple therapies and is expected to be the third leading cause of cancer-related death by 2025 [2]. The rapid progression and resistance to chemotherapy remain obstacles to improving prognosis, and the mechanisms are unclear [3].

To uncover the mechanisms of progression and chemoresistance in PC, we established 15 gemcitabine-sensitive patient-derived xenografts (PDXs) and 13 gemcitabine-resistant PDXs [4]. Through multi-omics analysis of these PDXs, we constructed a chemoresistance-related regulatory network. In this network, PRKRA, a protein activator of interferon-induced protein kinase EIF2AK2 was at the hub node, which provided important clues for further exploring the role of PRKRA in chemoresistance and disease progression [4].

PRKRA encodes the protein PACT, which plays a vital role in cellular stress and inflammation [5]. PRKRA functions as a cellular modulator via two pathways: on the one hand, PRKRA is phosphorylated at Ser287 under environmental stimuli and phosphorylates eukaryotic translation initiation factor 2 alpha kinase 2 (EIF2AK2). Phosphorylated EIF2AK2 can further phosphorylate eukaryotic translation initiation factor 2α (eIF2α), thereby inhibiting protein translation and enabling cells to adapt to environmental stress [5]. On the other hand, PRKRA can bind to Dicer and double-stranded RNA and participate in the maturation of microRNA precursors, thus affecting the expression of microRNA target genes [6]. It has been proven that PRKRA mutation is associated with dystonia, but very few studies have been reported on cancer [7]. Wang et al. found that lncRNA AL033381.2 could bind to PRKRA and upregulate the expression of PRKRA, thereby promoting liver cancer progression [8]. PRKRA was also found to promote chemoresistance in ovarian cancer by downregulating miRNA-515-3p and upregulating AXL [9]. However, the effects of PRKRA on PC have not been reported.

NF-κB pathway is involved in the response of cells to various stimuli, such as inflammatory factors, free radicals, ultraviolet rays, radiation, bacteria, or viruses [10]. Previous studies have shown that the NF-κB pathway could promote the proliferation, invasion, metastasis, and angiogenesis of PC and regulate the chemoresistance of PC [[11], [12], [13]]. However, the relationship between PRKRA and the NF-κB pathway was unknown in PC.

MMP1 (matrix metalloproteinase 1) is a member of the MMP family, which play a role in the degradation of extracellular matrix and regulate the biological behavior of cancer cells [14,15]. MMP1 could promote the progression of colorectal cancer by epithelial-mesenchymal transition (EMT) and PI3K/Akt/c-myc pathway [16,17]. But the role of MMP1 in PC remains further exploration.

The present study was designed to investigate the relationship between PRKRA and PC clinicopathologic features and chemosensitivity. The effects of PRKRA on cancer progression and chemoresistance were validated in vitro and in vivo. The potential mechanisms of PRKRA promoting PC were also explored. This study highlighted the oncogenic effects of the PRKRA/NF-κB/MMP1 axis in PC and targeting this axis may be a promising strategy for improving prognosis.

## 2 Materials and methods

### 2.1 Data collection and processing

RNA-seq data in the TCGA database were processed by Toil and downloaded from XENA (https://xenabrowser.net/datapages/). 177 TCGA pancreatic adenocarcinoma (PAAD) samples and 4 TCGA normal adjacent samples were included. The clinical and overall survival (OS) data of 177 PAAD patients were obtained from the TCGA database. PAAD patients without clinical information and duplicate samples were removed. Log2 transformations were performed for TPM (transcripts per million reads) of RNA-seq data.

Microarray data of PDAC patients were obtained from the GEO database (http://www.ncbi.nlm.nih.gov/geo/). GSE71729 included 145 PDAC samples and 46 noncancerous samples. GSE28735 included 45 matching pairs of pancreatic tumors and adjacent non-tumor tissues.

### 2.2 Immunohistochemistry (IHC)

Tumors and paired adjacent tissues were obtained from 334 surgical specimens. Written informed consent was obtained from all patients. Tissues were fixed with formalin and embedded in paraffin. Rabbit anti-human PRKRA polyclonal antibody (10771-1-AP, Proteintech, 1:200) and Rabbit anti-human MMP1 polyclonal antibody (10371-2-AP, Proteintech, 1:200) were used for the immunohistochemical staining. The expression of PRKRA and MMP1 in ductal adenocarcinoma cells and normal ductal cells were evaluated. The results of immunohistochemistry were assessed according to the percentage of positive cells and intensity of staining respectively as described previously [18].

### 2.3 Cell culture

The PC cell lines AsPC-1 and MiaPaCa-2 were cultured in RPMI 1640 (01-100-1ACS, Biological Industries, Israel) and DMEM-high glucose (01-052-1ACS, Biological Industries, Israel) medium respectively, which were supplemented with 10% fetal bovine serum (04-001-1ACS, Biological Industries, Israel) and incubated in a 5% CO_2_ at 37 °C. Cells were passaged at the ratio of 1:3 when the confluence reached 80%–90% using trypsin (03-045-1B, Biological Industries, Israel).

### 2.4 siRNA and plasmid transfection

SiRNA for PRKRA and normal control siRNA were provided by Guangzhou RiboBio Company. The sequence of siRNA targeting PRKRA was GTAAGAAGCTGGCGAAACA (Si-PRKRA-1) and GCTAGAGTCATTTATGGAA (Si-PRKRA-2). Full-length of PRKRA cDNA was subcloned into a GV702 vector to construct PRKRA-overexpression plasmid. Transient transfections of siRNA and plasmid were realized by Lipofectamine 8000 (C0533, Beyotime, China) according to the instruction of the manufacturer.

### 2.5 Knockout of PRKRA by CRISPR/Cas9

For stable knockout of PRKRA, AsPC-1 cells were infected with lentivirus expressing Cas9 and selected with puromycin for 7 days. Then, AsPC-1 cells expressing Cas9 were infected with sgRNA lentivirus and selected with neomycin for 7 days. The sequences of sgRNA are as follows: sgRNA-1: GTCACCAACGGTTACTCTGA; sgRNA-2: GGCGAAACATAGAGCTGCAG; sgRNA-NC: CGCTTCCGCGGCCCGTTCAA.

### 2.6 RT-qPCR

TRIzol reagent (Invitrogen, USA) was used for total RNA extraction. cDNA synthesis was performed using the HiScript III 1st Strand cDNA Synthesis Kit (R312-01, Vazyme, China) and qPCR was performed using Taq Pro Universal SYBR qPCR Master Mix (Q712-02, Vazyme, China). The primers for PCR are listed in Supplementary Table S1.

### 2.7 Western blot

Western blot was performed as previously described [19]. PC cells were harvested by RIPA buffer (P0013B, Beyotime, China) according to the manufacturer’s instructions. 20 μg total proteins were separated by SDS-PAGE and transferred to PVDF membranes. Then, primary antibodies against PRKRA (10771-1-AP, Proteintech, USA), MMP1 (10371-2-AP, Proteintech, USA), pNF-κB (3033, Cell Signaling Technology, USA), β-actin (AF0003, Beyotime, China) and secondary antibodies were used to bind to target protein bands.

### 2.8 Transcriptome sequencing and data analysis

AsPC-1 cells with PRKRA knockdown and overexpression were harvested for transcriptome sequencing. The total RNA was extracted by using RNeasy MINI KIT. An Illumina platform was applied for the directional libraries generation. Cuffdiff was used to obtain the sequence count data [fragments per kilobase of transcript per million mapped reads (FPKM) values] to conduct differential expression analysis. Biomarker Biotechnology Corporation performed the library construction and sequencing.

### 2.9 Proliferation and chemosensitivity assay

Cells were seeded into 96-well plates at 2000 cells/well for proliferation assay. Cell Counting Kit-8 (CCK-8) reagent (Dojindo, Japan) was added into 96-well plates at 0, 24, 48, and 72 h after seeding, and was incubated for an additional 2.5 h at 37 °C. Cells were seeded at 4000 cells/well for chemosensitivity assay. Cells were treated with gemcitabine at the concentrations of 10 nM, 100 nM, 1 μM, 10 μM, and 100 μM for 48 h before the CCK-8 reagent was added. The optical density (OD) at 450 nm was measured by the microplate reader.

### 2.10 Migration and invasion assay

Cell migration assay was performed as described previously [20]. In brief, 100,000 AsPC-1 cells or 80,000 MiaPaCa-2 cells in medium without fetal bovine serum were placed into the upper chamber of a Transwell unit (3422, Corning, USA), and medium with 10% fetal bovine serum was added in the lower chamber. After 24 h, the migrated cells were fixed and stained with crystal violet. For invasion assay, the polycarbonate membranes of the upper chambers were coated with Matrigel (354234, Corning, USA) diluted at a 1:10 ratio with the medium before use.

### 2.11 Xenograft models

A protocol was prepared before the study without registration. The sample size calculation was based on a similar experiment we conducted previously [21]. All animal experiments were conducted under the ethical guidelines of the Peking Union Medical College ethics committee (Beijing, China). AsPC-1 cells (5 × 10^6^) with knockout of PRKRA were suspended in PBS and subcutaneously injected into BALB/c nude mice (5 weeks old, female, 18 ± 2 g). Mice were kept in polyacrylic cages with a temperature: 22 ± 2 °C, humidity: 50% ± 5%, 12 h light/dark cycle, and sufficient water and food. The injection was performed by experienced investigators to minimize the animal pain and unnecessary death. Tumor size was measured every 3 days and calculated by the formula: volume (mm^3^) = 1/2(width)^2^ × length. The mice were sacrificed 30 days after cell injection and all of the mice were included in the analysis.

### 2.12 Culture of PC organoid

The resected PC samples were digested using collagenase type II (5 mg/ml) and DNase I (100 μg/ml) for 1 h. The supernatant was centrifuged at 300 g, 4 °C for 5 min and the harvested cells were washed twice with medium and embedded in Matrigel (354234, Corning, USA) in 24-well plates. After solidification in a 37 °C incubator for 30 min, the organoid complete medium was added. The organoid complete medium consisted of Wnt-3a (50 ng/ml), R-spondin 1 (100 ng/ml), B27 supplement (1×), Nicotinamide (10 mM), N-acetylcysteine (1.25 mM), Noggin (100 ng/ml), EGF (50 ng/ml), FGF-10 (100 ng/ml), Gastrin I (10 nM), A83-01 (500 nM), Y-27632 (10.5 μM) and PGE2 (1 μM).

### 2.13 Chromatin immunoprecipitation (CHIP)

ChIP was performed using BeyoChIP™ Enzymatic ChIP Assay Kit (P2083S, Beyotime, China) according to the manufacturer's instructions. Anti-P65 antibody (10745-1-AP, Proteintech, USA) was applied to immunoprecipitate protein from AsPC-1 and MiaPaCa-2 cell lysates with A/G magnetic beads conjugated to the anti-P65 antibody. qPCR was performed to analyze the enrichment of MMP1. Primers used for ChIP-qPCR are listed in Supplementary Table S1.

### 2.14 Dual-luciferase reporter assay

The plasmids with different length or mutant form of MMP1 promoter region and wild-type form of MMP1 promoter region was constructed based on the GV238 backbone. For the luciferase assay, PC cells were plated in 12-well plates and co-transfected with a dual-luciferase reporter and PRKRA overexpression plasmid by Lipofectamine 8000 (C0533, Beyotime, China) according to the manufacturer's instruction. Luciferase activity was measured by Dual-Luciferase Assay (11402ES60, YEASEN, China). The relative luciferase activity was defined as Renilla luciferase activity against Firefly luciferase activity.

### 2.15 Ethical statement

The authors are accountable for all aspects of the work in ensuring that questions related to the accuracy or integrity of any part of the work are appropriately investigated and resolved. The study was conducted in accordance with the Declaration of Helsinki (as revised in 2013). The study was approved by the Ethics Committee of Peking Union Medical College Hospital (NO: JS2568). Written informed consent was obtained from all patients. Animal experiments were performed under a project license (NO: DW-2022-155) granted by the Ethics Committee of Peking Union Medical College Hospital, in compliance with institutional guidelines for the care and use of animals.

### 2.16 Statistical analysis

Chi-squared test was used for categorical variables and the Wilcoxon rank sum test for continuous variables. Survival analysis was conducted using the Kaplan-Meier method and compared using the log-rank test. Variables with P < 0.20 in the univariate analysis were selected for multivariate analysis. P < 0.05 was regarded as statistically significant. The SPSS 22.0 software (Chicago, IL) and GraphPad Prism 8 (La Jolla, CA) were used for data analysis.

## 3 Results

### 3.1 Upregulated PRKRA in cancer correlated with poor prognosis of PC

The results of IHC showed that PRKRA was upregulated in pancreatic ductal adenocarcinoma cells than paired normal pancreatic ductal cells in specimens from patients (Fig. 1A). We then compared the expression of PRKRA in PC and normal tissues in the TCGA database, and it was shown that PRKRA was significantly overexpressed in cancer (Fig. 1B). The expression of PRKRA in the histological G1 grade group was lower than that in G2, G3, and G4 groups (Fig. 1C). The overexpression of PRKRA in PC was further confirmed with GEO DataSets GSE71729 (Fig. 1D). To investigate the prognostic value of PRKRA in PC, the KM plotter indicated that the overall survival was much better in the PRKRA-low group in both the clinical cohort [HR = 1.61 (1.03–2.53), P = 0.008] and TCGA database [HR = 1.76 (1.16–2.67), P = 0.008] (Fig. 1E and F). Table 1 showed that there was a significant difference in histological grade between high and low-expression groups, indicating that PRKRA may serve as the potential biomarker for prognosis.

**Fig. 1:**
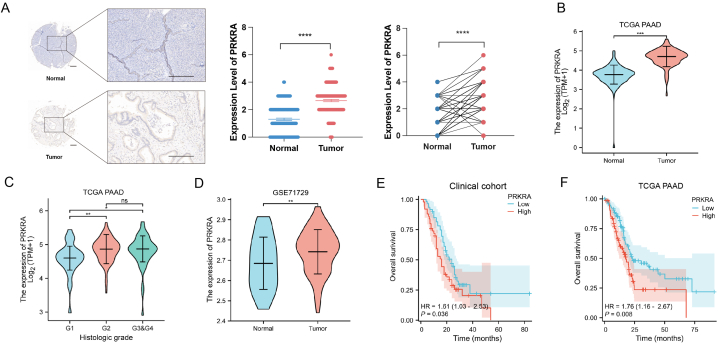
PRKRA was upregulated in PC and related to poor prognosis. (A) The expression of PRKRA in PC and normal tissues in IHC. (B) The expression of PRKRA in PC and normal tissues in the TCGA database. (C) The expression of PRKRA in patients with different histological grades. (D) The expression of PRKRA in PC and normal tissues in the GEO dataset. (E–F) Overall survival between the high and low PRKRA expression groups in the clinical cohort and TCGA. (Scale bar = 200 μm; *p value < 0.05; **p value < 0.01; ***p value < 0.001; ****p value < 0.0001.)

**Table 1:** Correlation between PRKRA expression and clinical characteristic.

Characteristic	Low	High	p
n	102	75	
Age, n (%)			0.212
≤65	49 (27.7%)	44 (24.9%)	
>65	53 (29.9%)	31 (17.5%)	
Gender, n (%)			0.464
female	49 (27.7%)	31 (17.5%)	
male	53 (29.9%)	44 (24.9%)	
TNM_pathologic_T, n (%)			0.793
T1	5 (2.9%)	2 (1.1%)	
T2	15 (8.6%)	9 (5.1%)	
T3	78 (44.6%)	63 (36%)	
T4	2 (1.1%)	1 (0.6%)	
TNM_pathologic_N, n (%)			0.432
N0	31 (18%)	18 (10.5%)	
N1	68 (39.5%)	55 (32%)	
TNM_pathologic_M, n (%)			0.329
M0	41 (23.2%)	38 (21.5%)	
M1	2 (1.1%)	2 (1.1%)	
MX	59 (33.3%)	35 (19.8%)	
Pathologic stage, n (%)			0.808
Stage I	14 (8%)	7 (4%)	
Stage II	82 (47.1%)	64 (36.8%)	
Stage III	2 (1.1%)	1 (0.6%)	
Stage IV	2 (1.1%)	2 (1.1%)	
Histological grade, n (%)			**0.002**
G1	26 (14.9%)	5 (2.9%)	
G2	48 (27.4%)	46 (26.3%)	
G3	24 (13.7%)	24 (13.7%)	
G4	2 (1.1%)	0 (0%)	

### 3.2 PRKRA promoted proliferation, migration, invasion chemoresistance of PC cells in vitro

**Fig. 2:**
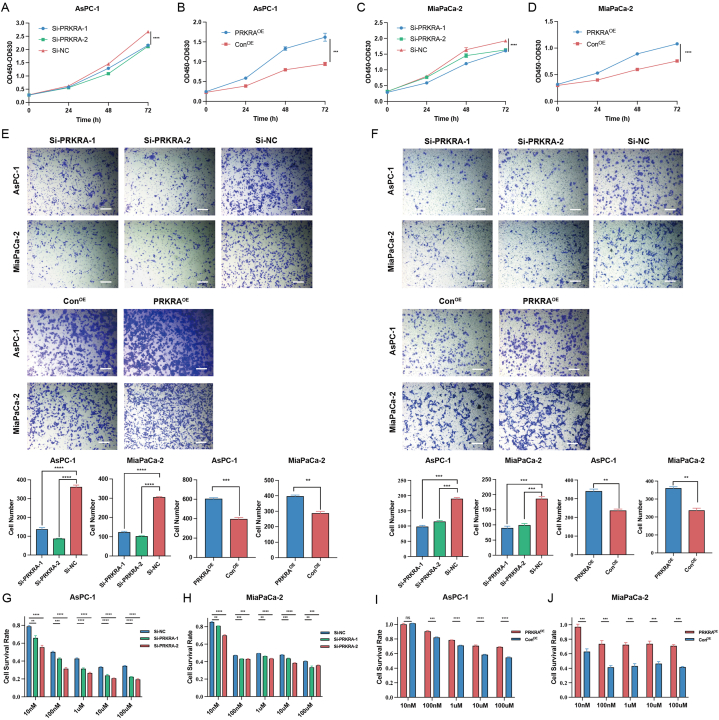
The effects of PRKRA on the biological phenotype of PC cells. (A–D) The proliferation of AsPC-1 and MiaPaCa-2 cells after PRKRA knockdown and overexpression was detected by CCK-8 assay. (E–F) The migration and invasion capacity of PC cells was demonstrated by Transwell assay. (G–J) The survival rates of AsPC-1 and MiaPaCa-2 cells treated with different concentrations of gemcitabine were calculated based on the CCK-8 assay. (Scale bar = 100 μm; *p value < 0.05; **p value < 0.01; ***p value < 0.001; ****p value < 0.0001.)

SiRNA and overexpression plasmids were applied to regulate PRKRA. The knockdown and overexpression of PRKRA were verified by RT-qPCR and Western blot (Fig. S1). CCK-8 assay was applied to evaluate the effect of PRKRA on proliferation. The growth rates of AsPC-1 and MiaPaCa-2 cells were significantly decreased after the downregulation of PRKRA and increased with the overexpression of PRKRA (Fig. 2A–D). Similarly, the migration and invasion capacity of PC cells also decreased or increased after the knockdown and overexpression of PRKRA (Fig. 2E and F). The sensitivity of AsPC-1 and MiaPaCa-2 cells to gemcitabine was increased after the knockdown of PRKRA and increased after overexpression of PRKRA (Fig. 5G–J).

### 3.3 PRKRA facilitated the growth of PC organoids and mice xenograft models

**Fig. 3:**
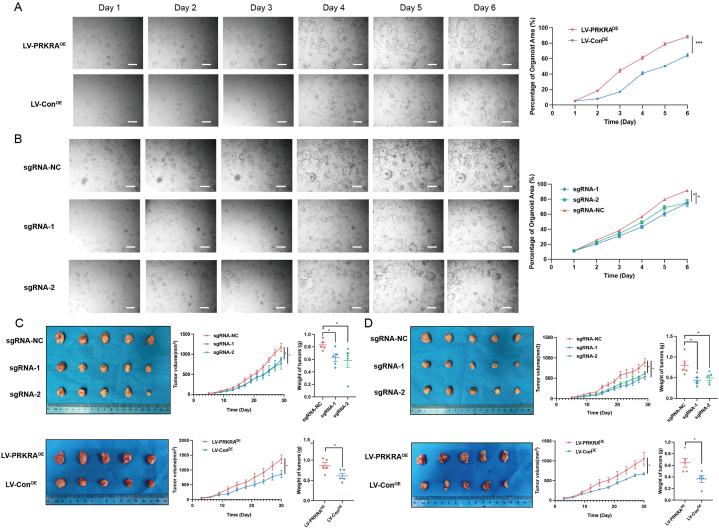
The effects of PRKRA on PC organoids and mice subcutaneous xenograft models. (A–B) Photographs of PC organoids with PRKRA overexpression and knockout; the percentages of organoids' area were shown in the right panel. (C) Photographs of dissected subcutaneous tumors of PRKRA overexpression and knockout groups; the growth curves and weights of tumors were shown in the right panel. (D) Photographs of dissected subcutaneous tumors of PRKRA overexpression and knockout groups when treated with gemcitabine; the growth curves and weights of tumors were shown in the right panel. (Scale bar = 200 μm; *p value < 0.05; **p value < 0.01; ***p value < 0.001; ****p value < 0.0001.)

The established PC organoids were validated by IHC, and the comparison of PC-related markers between primary tumor tissue and organoids was provided in Fig. S2. Organoids with PRKRA knockout and overexpression were established by CRISPR/Cas9 and lentivirus. The result showed that the growth rate of organoids with PRKRA knockout was significantly lower than that of the control group. And the proliferation of organoids increased after PRKRA overexpression (Fig. 3A and B). To evaluate the effects of PRKRA on PC progression in vivo, AsPC-1 cells with PRKRA knockout and overexpression were established. The tumor growth rates significantly decreased after PRKRA knockout and increased after PRKRA overexpression (Fig. 3C). When treated with gemcitabine, the tumor growth was inhibited more obviously in the PRKRA knockout group than in the control group and promoted in the PRKRA overexpression group (Fig. 3D).

### 3.4 PRKRA promoted the progression of PC via MMP1

**Fig. 4:**
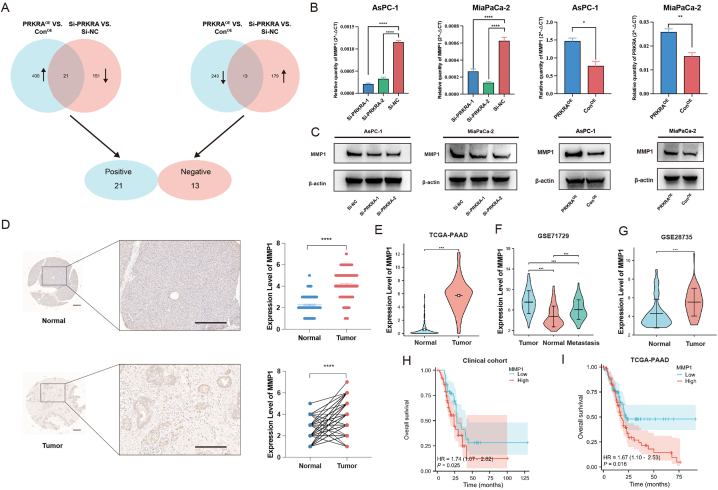
Regulation of MMP1 by PRKRA and clinical significance of MMP1. (A) The Venn analysis of differentially expressed genes between two groups. (B–C) RT-qPCR and Western blot validation of MMP1 expression after PRKRA knockdown and overexpression. (D) IHC analysis of MMP1 expression in clinical samples. (E–G) Analysis of MMP1 expression in TCGA and GEO datasets. (H–I) Overall survival between the high and low MMP1 expression groups in the clinical cohort and TCGA. (Scale bar = 200 μm; *p value < 0.05; **p value < 0.01; ***p value < 0.001; ****p value < 0.0001.)

To further explore the mechanisms of cancer promotion by PRKRA, transcriptome RNA-sequencing was applied. AsPC-1 cells with PRKRA knockdown or overexpression were harvested. In AsPC-1 cells with PRKRA knockdown, there were 192 upregulated and 172 downregulated genes compared with normal control (|log_2_FC| ≥ 1 and p < 0.05). In AsPC-1 cells with PRKRA overexpression, there were 421 upregulated and 256 downregulated genes compared with normal control (|log2FC| ≥ 1 and p < 0.05). The differentially expressed gene lists were provided in supplementary materials. To further narrow down the genes related to PRKRA expression, the Venn analysis was performed. 21 genes positively and 13 genes negatively related to the expression of PRKRA (Fig. 4A). List of these genes was provided in Table S4. Among these genes, MMP1 was positively correlated with PRKRA expression, and involved in cancer progression and chemoresistance. Both RT-qPCR and Western blot confirmed that knockdown of PRKRA decreased MMP1 expression while overexpression of PRKRA increased MMP1 expression (Fig. 4B and C).

**Fig. 5:**
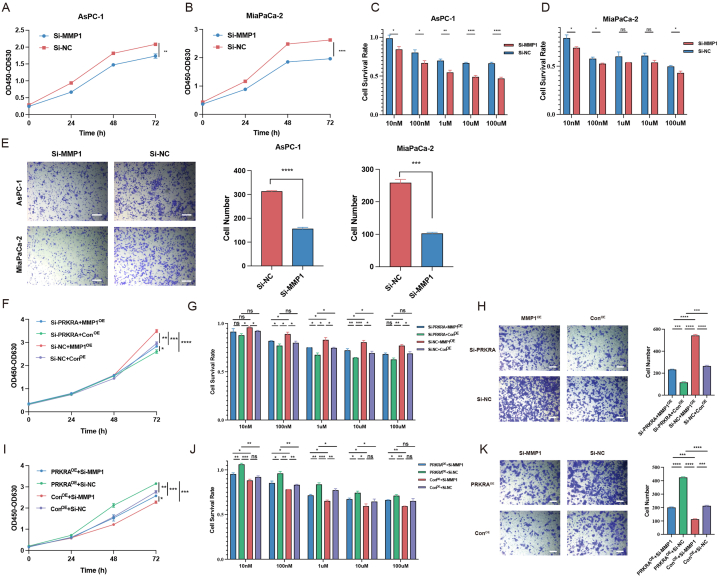
The effects of MMP1 on PC cells and rescue experiment. (A–B) The proliferation of AsPC-1 and MiaPaCa-2 cells after MMP1 knockdown was detected by CCK-8 assay. (C–D) The survival rates of AsPC-1 and MiaPaCa-2 cells treated with different concentrations of gemcitabine were calculated based on the CCK-8 assay. (E) The migration capacity of PC cells was demonstrated by the Transwell assay. (F–H) MMP1 overexpression in PRKRA knockdown cells rescued the proliferation, chemoresistance, and migration. (I–K) The proliferation, chemoresistance, and migration were decreased by Si-MMP1 in PRKRA overexpression cells. (Scale bar = 100 μm; *p value < 0.05; **p value < 0.01; ***p value < 0.001; ****p value < 0.0001.)

In the IHC of clinical samples, MMP1 was overexpressed in pancreatic ductal adenocarcinoma cells than paired normal pancreatic ductal cells (Fig. 4D). Similarly, in TCGA and GEO datasets, MMP1 was also upregulated in tumor tissues and metastasis (Fig. 4E–G). Besides, the expression levels of MMP1 were related to patients' prognosis in the clinical cohort and TCGA dataset (Fig. 4H and I).

To further investigate the effects of MMP1 on PC cells, we applied siRNA to downregulate MMP1 expression in PC cells. The results showed that the knockdown of MMP1 decreased the proliferation, chemoresistance, and migration of PC cells (Fig. 5A–E). Then, we performed the rescue experiment where MMP1 was upregulated in PRKRA knockdown PC cells and downregulated in PRKRA overexpression PC cells. The results showed that the decreased proliferation, chemoresistance, and migration caused by Si-PRKRA could be rescued by MMP1 upregulation, while the increased proliferation, chemoresistance, and migration caused by PRKRA overexpression could be decreased by Si-MMP1 (Fig. 5F–K). These results indicated that PRKRA promoted PC progression via MMP1, and MMP1 also acted as an oncogenic gene in PC.

### 3.5 PRKRA regulated expression of MMP1 by NF-κB pathway

**Fig. 6:**
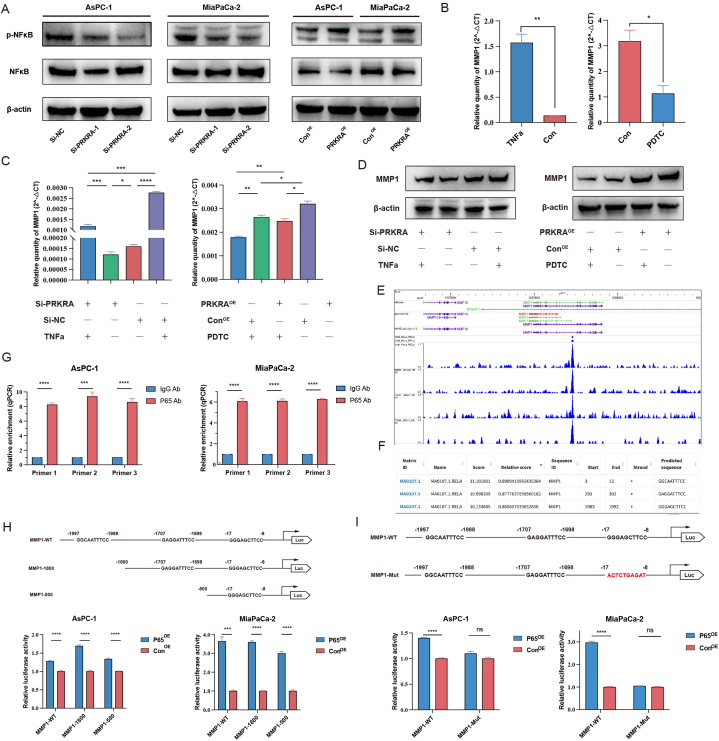
Regulation of MMP1 expression by PRKRA via NF-κB pathway. (A) The phosphorylation of NF-κB was detected by Western blot after PRKRA knockdown and overexpression. (B) The changes in MMP1 mRNA levels after treatment with TNFα and PDTC were detected by RT-qPCR. (C–D) The MMP1 mRNA and protein levels could be recused by TNFα and PDTC after PRKRA knockdown or overexpression. (E–F) Cistrome and JASPAR database showed potential binding of P65 to the MMP1 promoter region. (G) ChIP was performed with an anti-P65 antibody in AsPC-1 and MiaPaCa-2 cells. (H–I) Dual luciferase assays were applied to confirm the binding site of P65 to the MMP1 promoter region in AsPC-1 and MiaPaCa-2 cells. (*p value < 0.05; **p value < 0.01; ***p value < 0.001; ****p value < 0.0001.)

Previous studies showed that PRKRA may participate in NF-κB pathway activation in the inflammatory response, and the NF-κB pathway also played a role in PC progression and chemoresistance [22,23]. Therefore, we hypothesized that PRKRA regulated MMP1 expression via the NF-κB pathway. The results of western blot showed that the phosphorylation level of NF-κB decreased after PRKRA knockdown and increased after PRKRA overexpression (Fig. 6A). When treated with TNFα, an agonist of NF-κB, the mRNA and protein levels were increased in PC cells. Meanwhile, the inhibitor of NF-κB, Pyrrolidine dithiocarbamate (PDTC) decreased the levels of MMP1 expression (Fig. 6B). To validate that PRKRA regulated MMP1 expression via the NF-κB pathway, we performed a rescue experiment. It showed that TNFα could rescue the expression of MMP1 decreased by Si-PRKRA, while PDTC decreased the expression of MMP1 induced by PRKRA overexpression plasmid (Fig. 6C and D). These results indicated that MMP1 expression was regulated by the NF-κB pathway.

As NF-κB pathway exerts effects on cells by nuclear translocation of subunits, such as P65 and P50, to enhance the transcription of target genes. We speculated that NF-κB serves as a transcriptional factor to promote the transcription of MMP1. To verify our hypothesis, we used the Cistrome ChIP-seq database to find that P65 was enriched at the promoter region of MMP1 in HeLa cells, KB cells, LNCaP cells, and MCF-7 cells (Fig. 6E). Then, we analyzed the promoter region of MMP1 (−2000 bp to +143 bp), and find three potential P65 binding sites (Fig. 6F). To validate the binding of P65 to the MMP1 promoter, ChIP-qPCR was performed. The results showed that the P65 binding elements immunoprecipitated by the P65 antibody in the MMP1 promoter were significantly enriched compared with the control antibody (Fig. 6G). To further determine the binding site of P65 to the MMP1 promoter, we constructed three luciferase reporter plasmids with different lengths of MMP1 promoter: pMMP1-WT, pMMP1-1800, and pMMP1-500. Dual luciferase assays showed that the relative activities of luciferase all significantly increased when P65 was upregulated, indicating that the binding site of P65 may be located at the −500bp of the MMP1 promoter region (Fig. 6H). Then, we constructed luciferase reporter plasmid with the mutant promoter of MMP1 (pMMP1-Mut), and the dual luciferase assays showed that the relative activities of luciferase did not change significantly after P65 overexpression (Fig. 6I). These results showed that NF-κB promoted the transcription of MMP1 by binding to the promoter region of MMP1.

### 3.6 PRKRA/MMP1 axis predicted prognosis and chemosensitivity in PC

**Fig. 7:**
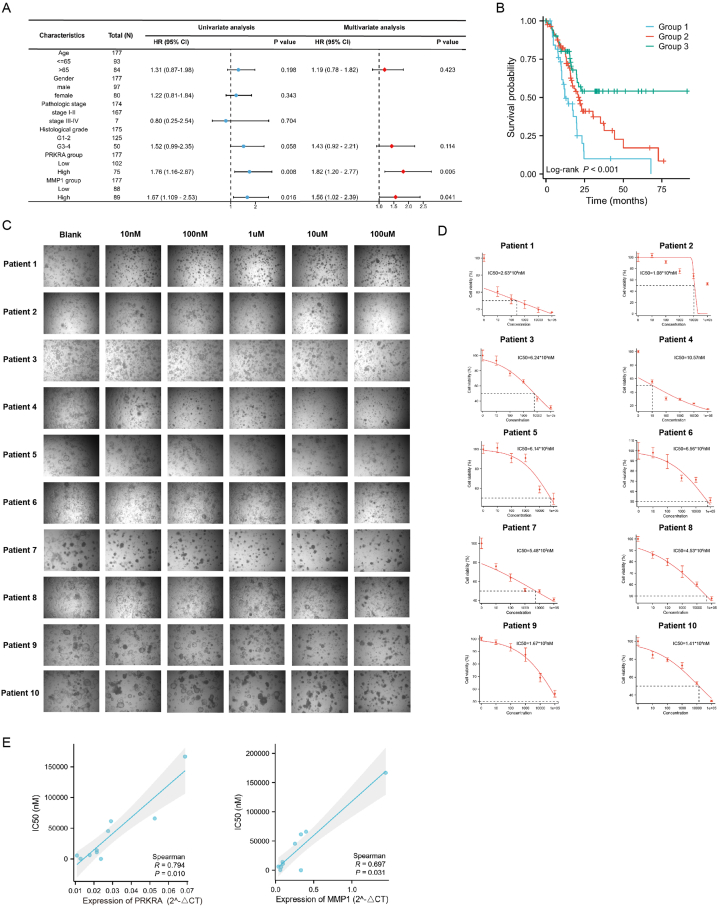
Values of PRKRA/MMP1 axis in prognosis and chemosensitivity. (A) Univariate and multivariate analysis of PRKRA/MMP1 axis and prognosis. (B) Comparison of prognosis in Group 1, Group 2, and Group 3. (C–D) Photographs and IC50 curves for gemcitabine sensitivity tests in organoids. (E) Correlation analysis between IC50 values and expression of PRKRA and MMP1.

To further explore the clinical values of the PRKRA/MMP1 axis in PC, we analyzed the TCGA dataset by using univariate and multivariate analysis. The results showed that PRKRA and MMP1 were independent risk factors for poor prognosis in PC (Fig. 7A). We divided patients into three groups based on PRKRA and MMP1 expression: Group 1 included patients with high expression of PRKRA and MMP1; Group 2 included patients with either PRKRA or MMP1 high expression; Group 3 included patients with low expression of PRKRA and MMP1. Then, Kaplan–Meier survival analysis demonstrated that the prognosis of patients in Group 1 was worse than Group 2 and 3, and the prognosis in Group 3 was best (Fig. 7B). The results indicated that PRKRA/MMP1 axis may serve as prognosis markers in PC.

Chemoresistance remains one of the challenges in the treatment of PC. To investigate the potential value of the PRKRA/MMP1 axis in predicting chemosensitivity, we applied PC organoids from 10 patients to test the sensitivity to gemcitabine and mRNA expression of PRKRA and MMP1. Photographs of organoids treated with different concentrations of gemcitabine were shown in Fig. 7C). The IC50 values were calculated for each organoid, and these IC50 values varied greatly, indicating that the sensitivity of different patients to gemcitabine has great heterogeneity (Fig. 7D). By Spearman analysis, we found that the IC50 values were positively related to the expression level of PRKRA and MMP1 (Fig. 7E).

## 4 Discussion

Although the prognosis of PC has improved in recent years, most patients suffer from tumor progression and chemoresistance [24]. To further uncover the mechanisms, we previously established PDX models, and transcriptome sequencing was applied to screen the key genes responsible for disease progression and chemoresistance [4]. PRKRA is one of the key genes and has been proven to regulate the proliferation of PC [4]. However, the role of PRKRA in cancer has rarely been investigated. Previous studies have confirmed that the mutation in the PRKRA gene is responsible for DYT16, a novel adulthood-onset dystonia, which accounts for 4.5% of all cases [25,26]. PRKRA participates in inflammation and the response to environmental stress, especially in viral infection [27,28]. The effects of PRKRA are partly realized by the activation of protein kinase R (PKR), an eIF2α kinase, which can activate eIF2α to inhibit translation and protein production [26]. In addition, PRKRA also participates in RNA silencing as a double-stranded RNA-binding protein, which can bind with Dicer [29]. PRKRA depletion can decrease the maturation of miRNA and reduce the efficiency of small interfering RNA-induced RNA interference [29]. The capacity of PRKRA to influence downstream pathways and miRNA maturation indicates that it may participate in cancer progression.

To determine the effects of PRKRA on the biological behavior of PC, we transfected PC cells with siRNA and overexpression plasmid. We found that PRKRA promoted the progression of PC in vitro and in vivo, suggesting that PRKRA may act as an oncogene in PC. Similarly, PRKRA also played a carcinogenic role in other tumors. For example, lncRNA AL033381.2 could bind to PRKRA and up-regulate the expression of PRKRA, thereby promoting the progression of liver cancer [8]. By binding to Dice, PRKRA downregulated the expression of miRNA-515-3p, leading to the up-regulation of target gene AXL and oxaliplatin resistance of ovarian cancer [9]. In addition, PRKRA promoted breast cancer metastasis through SUMOylation of Rac1 [30]. Our results are consistent with the results of these studies.

To further explore the underlying mechanisms, we applied transcriptome sequencing. We found that MMP1 expression was related to PRKRA expression. RT-qPCR, Western blot, and IHC also confirmed the regulatory effect of PRKRA on MMP1 expression. In the present study, we also found that the proliferation, migration, and resistance to gemcitabine of PC cells were decreased after MMP1 knockdown, which was consistent with the previous studies [[31], [32], [33]]. To verify that PRKRA regulates the biological behavior of PC through MMP1, we performed a rescue experiment and found that MMP1 could reverse the PRKRA-induced biological behavior changes in PC. Therefore, the regulatory role of the PRKRA/MMP1 axis in PC progression and chemoresistance was confirmed.

Previous studies mainly focused on the role of MMP1 in tumor invasion and metastasis, because of its ability of extracellular matrix degradation [17,34,35]. However, its role in chemoresistance has been rarely investigated. Considering that the promotion of invasion and metastasis by MMP1 is partly attributed to EMT which is involved in chemoresistance [36,37], it is reasonable that MMP1 promotes chemoresistance via EMT. Besides, MMP1 also increased stemness of cancer cells which may be another possible mechanism for MMP1-induced chemoresistance [32].

PRKRA plays a role in inflammation, and inflammation in tumors is closely related to disease progression [38,39]. The NF-κB pathway is an important part of PRKRA-induced inflammation, and it has been confirmed to be involved in the progression and chemoresistance of PC [40]. So we hypothesized that PRKRA regulated MMP1 via the NF-κB pathway. Treatment of PC cells with NF-κB agonists and inhibitors resulted in upregulation and downregulation of MMP1 mRNA and protein, suggesting that the NF-κB pathway regulated the transcription of MMP1. Since we have found enrichment of P65 at the MMP1 promoter region in the Cistrome database, and binding sites of P65 at the MMP1 promoter in the JASPAR database, we applied ChIP-qPCR for direct verification. After confirming by ChIP-qPCR that P65 was able to bind to the MMP1 promoter region, we also determined the specific binding site using dual luciferase reporter assays.

We further explored the prognostic value of PRKRA and MMP1 in PC and found that PRKRA and MMP1 were overexpressed in tumor tissues and correlated with poor prognosis. In addition, we also performed a joint analysis of PRKRA and MMP1 in the prognosis of PC patients, suggesting that patients with high expression of both have the worst prognosis.

However, there are some challenges in evaluating the role of PRKRA and MMP1 in predicting the chemosensitivity of PC. On the one hand, not all patients receive adjuvant chemotherapy with gemcitabine. On the other hand, the actual clinical effects require a long-term follow-up, and the sensitivity is difficult to quantify. So we used the organoid models. Organoids are three-dimensional cell cultures derived from stem cells. Compared with ordinary 2D cultured cells, organoids are more like the true tumor microenvironment in vivo, and can perfectly maintain the genetic characteristics of the original tumor with stable passage and expansion [41,42]. Several studies have shown that the drug sensitivity of organoids is highly consistent with the actual clinical situation, which means an organoid is a powerful tool in the individualized and precise treatment of PC [[43], [44], [45]]. Through the organoid model, we can test the sensitivity to gemcitabine in a short time. At the same time, we can collect the organoids cultured in the same period to detect the expression of PRKRA and MMP1 and analyze the correlation between their expression and drug sensitivity. The results showed that the expression levels of PRKRA and MMP1 were significantly correlated with the IC50 value of organoids, suggesting that PRKRA and MMP1 could be used as markers to predict the sensitivity to gemcitabine.

This study also has some limitations. For example, we demonstrated that PRKRA could activate the NF-κB pathway, but the exact mechanisms remain for further exploration. In addition, due to the long culture period of PC organoids, only 10 PC organoids were used in this study for drug tests. Therefore, the reliability of the conclusion is relatively weak, and the sample size needs to be further increased in the future.

## 5 Conclusions

Through the above experiments, we clarified the role of PRKRA in promoting the progression and chemoresistance of PC, and initially demonstrated the specific mechanism of PRKRA/NF-κB/MMP1 pathway. This pathway has potential value in the prognosis evaluation and chemotherapy sensitivity prediction of PC and can be a novel therapeutic target in the treatment of PC.

## Author contributions

Jiangdong Qiu: Performed the experiments; Analyzed and interpreted the data; Wrote the paper.

Mengyu Feng; Wenhao Luo: Performed the experiments.

Gang Yang; Jinxin Tao: Analyzed and interpreted the data.

Dan Su; Fangyu Zhao; Yueze Liu: Contributed reagents, materials, analysis tools or data.

Taiping Zhang: Conceived and designed the experiments.

## Data availability statement

Data associated with this study has been deposited at the TCGA database (https://portal.gdc.cancer.gov/), and GEO database (http://www.ncbi.nlm.nih.gov/geo/).

## Funding

This study was supported by grants from the Fundamental Research Funds for the Central Universities (3332022115); National High Level Hospital Clinical Research Funding (2022-PUMCH-D-001); National Natural Science Foundation of China (No. 81972258, No. 82103016, No. 82102958); CAMS Innovation Fund for Medical Sciences (CIFMS) (2021-1-I2M-002); National Multidisciplinary Cooperative Diagnosis and Treatment Capacity Building Project for Major Diseases and the Non-profit Central Research Institute Fund of Chinese Academy of Medical Sciences (2018PT32014).

## Declaration of competing interest

The authors declare that they have no known competing financial interests or personal relationships that could have appeared to influence the work reported in this paper

## Figures and Tables

**figs1:**
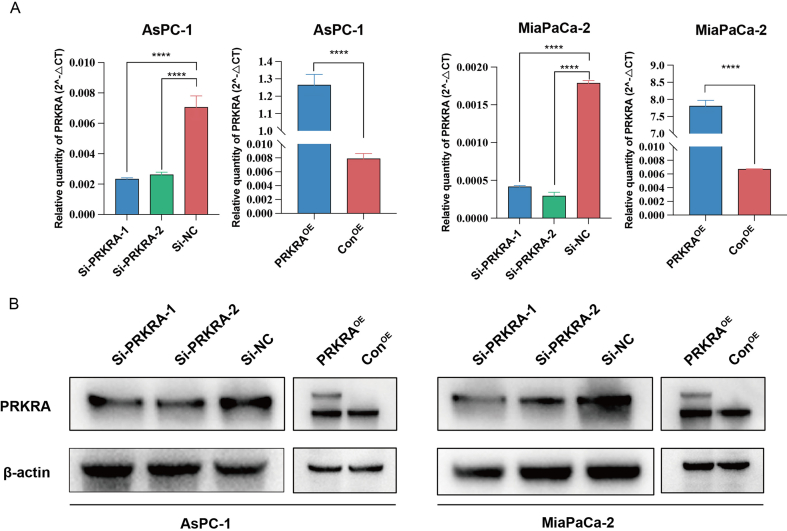


**figs2:**