# Supplementary material for: PRKRA promotes pancreatic cancer progression by upregulating MMP1 transcription via the NF-κB pathway
Source: Heliyon. 2023 Jun 10;9(6):e17194. doi: 10.1016/j.heliyon.2023.e17194 (PMC10361375; doi:10.1016/j.heliyon.2023.e17194)
Supplement: Multimedia component 4 [file mmc4.docx]

Table S4. List of differentially expressed genes after Venn analysis.

| Gene | Correlation | Gene | Correlation |
| --- | --- | --- | --- |
| PCDHGB7 | Positive | RTEL1-TNFRSF6B | Negative |
| SYS1-DBNDD2 | Positive | AC138811.2 | Negative |
| AC118553.2 | Positive | BCL2L2-PABPN1 | Negative |
| ARHGAP11B | Positive | AC004754.1 | Negative |
| MMP1 | Positive | TMEM151A | Negative |
| TCEA2 | Positive | AL662899.2 | Negative |
| ATXN7 | Positive | C1QTNF5 | Negative |
| AC020915.1 | Positive | UPK3BL1 | Negative |
| GNRH1 | Positive | RDM1 | Negative |
| PRAF2 | Positive | CCL15-CCL14 | Negative |
| PCDHGA10 | Positive | AC022384.1 | Negative |
| HTD2 | Positive | GVQW3 | Negative |
| TCEAL9 | Positive |  |  |
| ZNF350 | Positive |  |  |
| TOMM6 | Positive |  |  |
| MINOS1-NBL1 | Positive |  |  |
| RAB6B | Positive |  |  |
| RGL4 | Positive |  |  |
| ATP1A3 | Positive |  |  |
| HIC1 | Positive |  |  |
| RNFT2 | Positive |  |  |
