# Supplementary material for: PRKRA promotes pancreatic cancer progression by upregulating MMP1 transcription via the NF-κB pathway
Source: Heliyon. 2023 Jun 10;9(6):e17194. doi: 10.1016/j.heliyon.2023.e17194 (PMC10361375; doi:10.1016/j.heliyon.2023.e17194)
Supplement: Multimedia component 6 [file mmc6.pptx]

## Slide 1
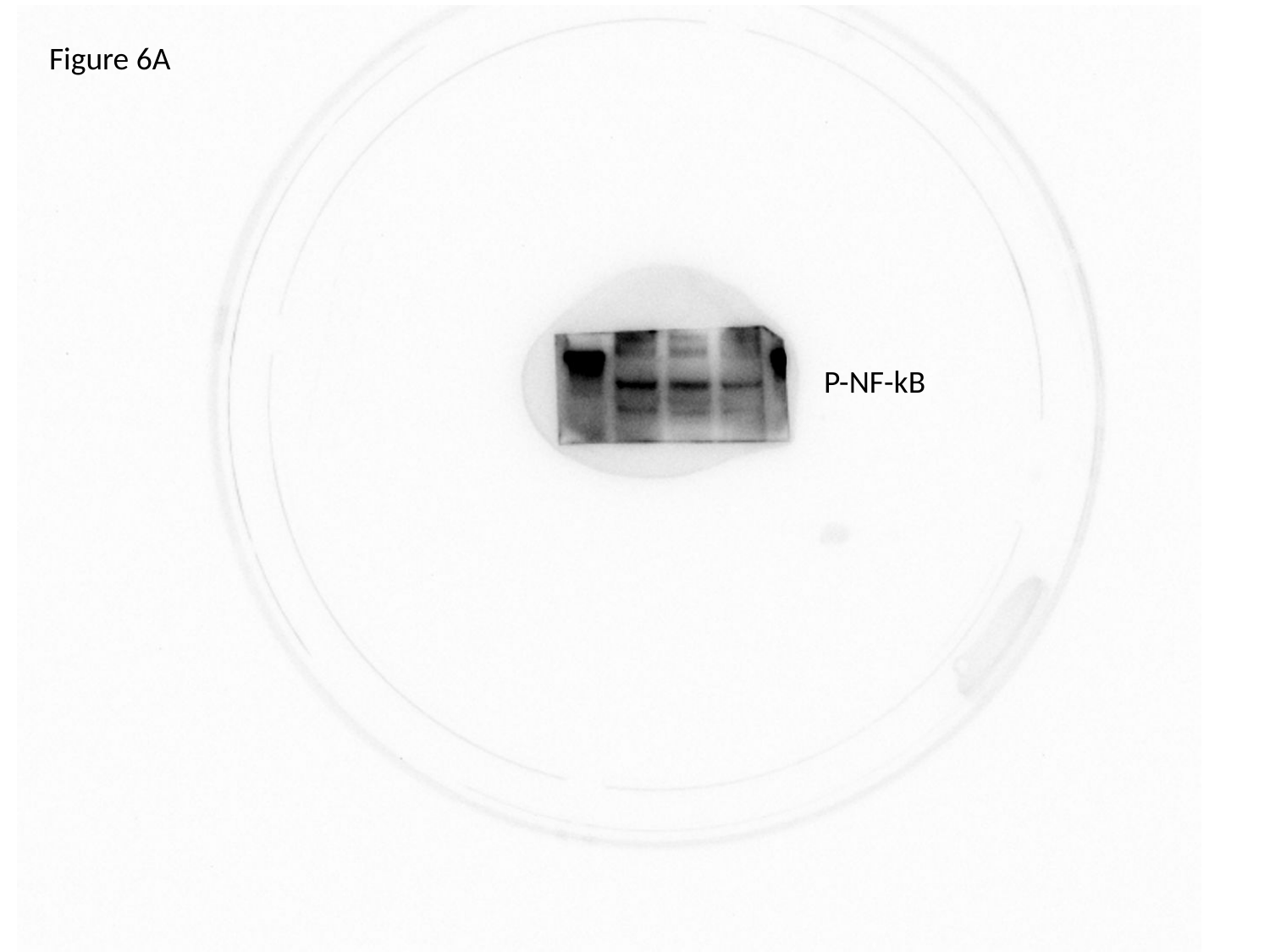

Figure 6A
P-NF-kB

## Slide 2
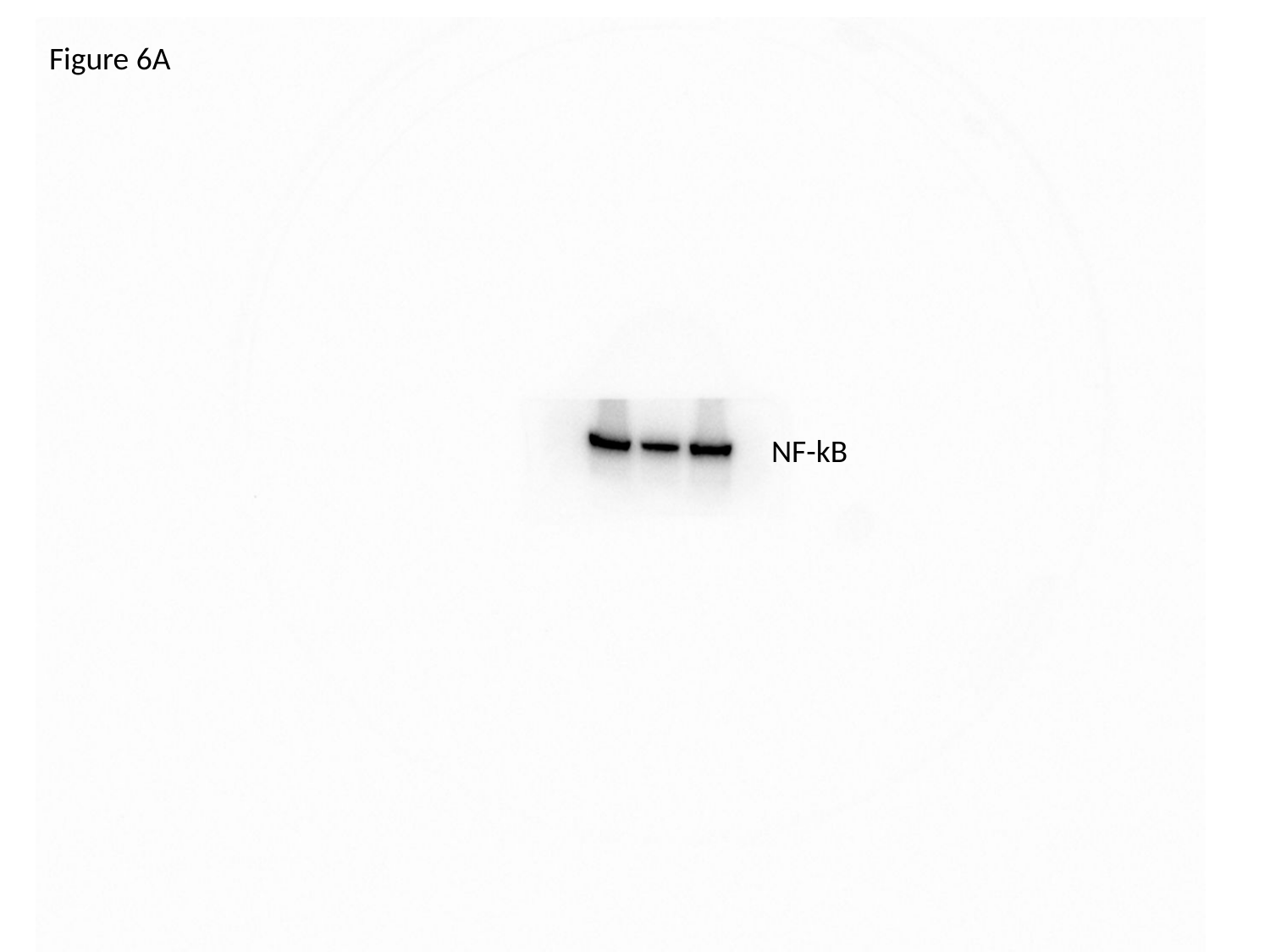

Figure 6A
NF-kB

## Slide 3
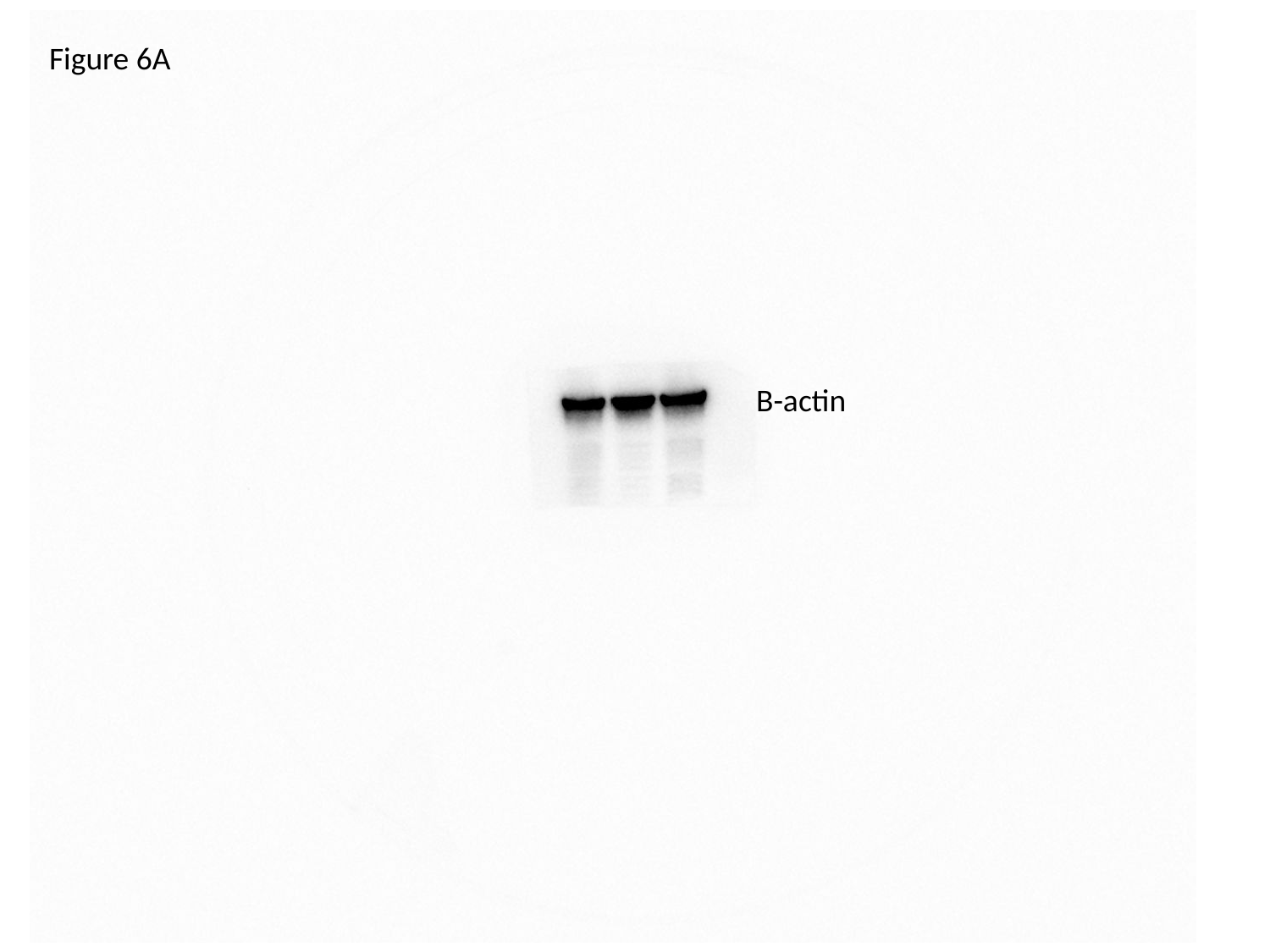

Figure 6A
B-actin

## Slide 4
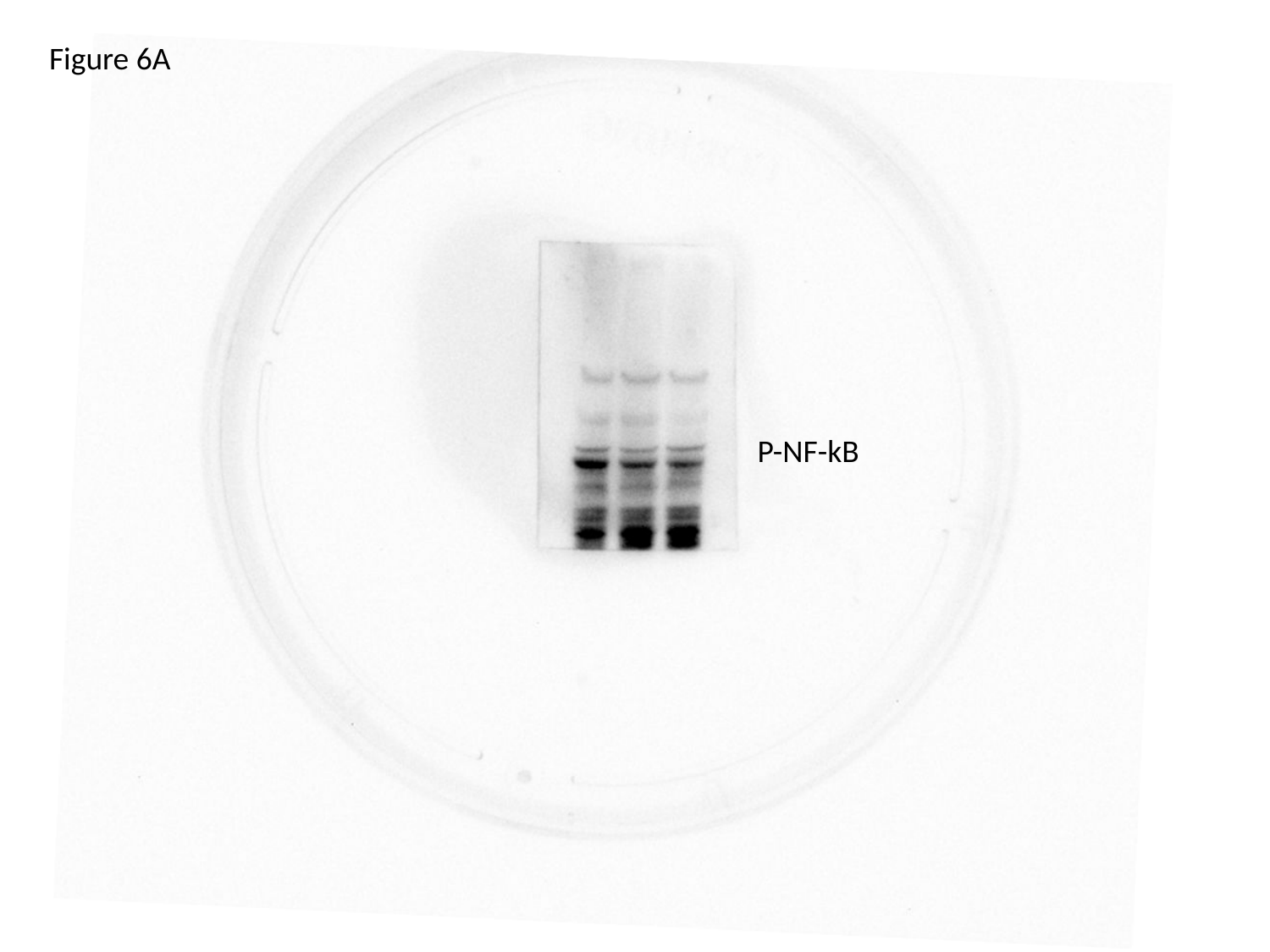

Figure 6A
P-NF-kB

## Slide 5
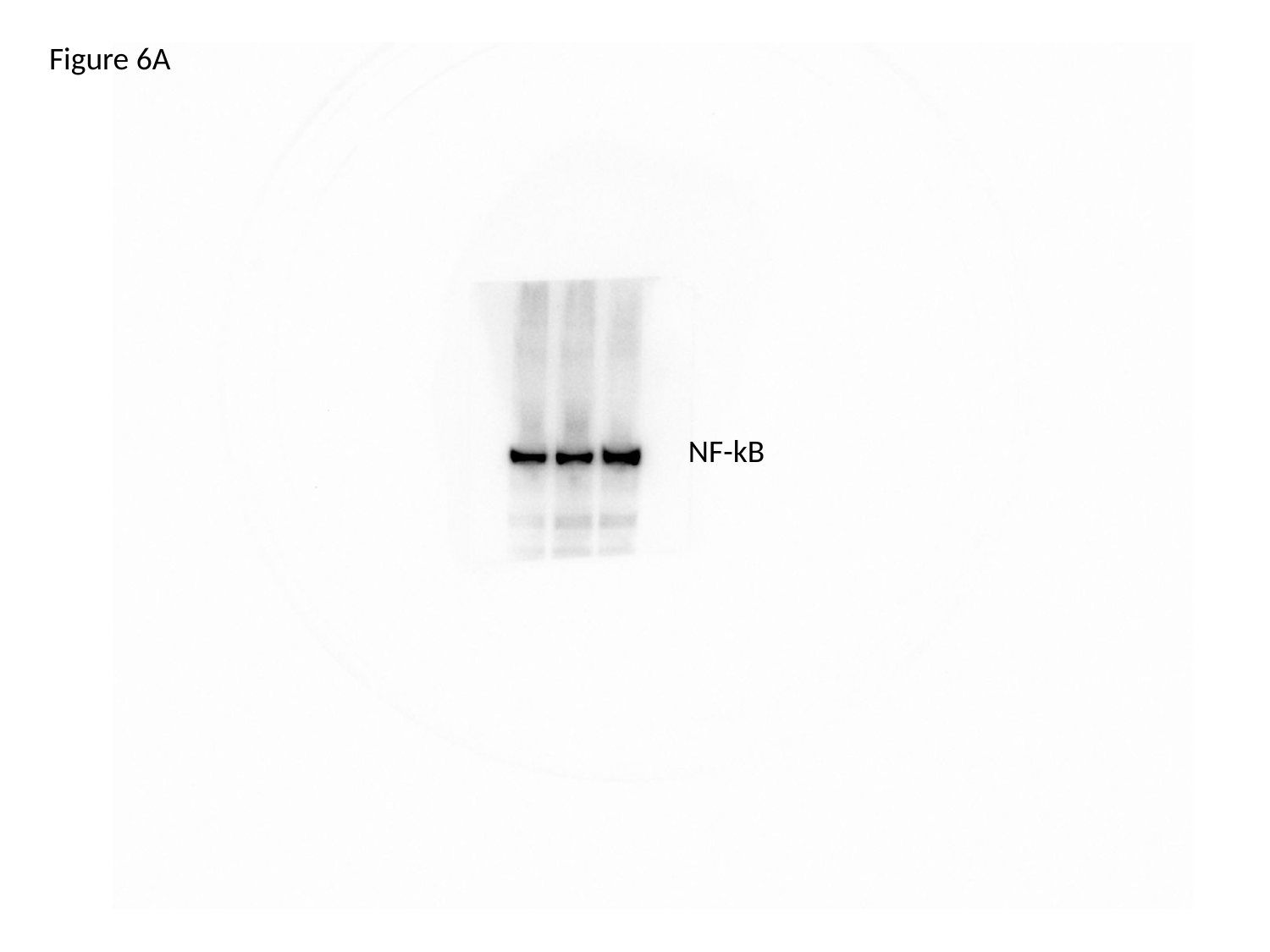

Figure 6A
NF-kB

## Slide 6
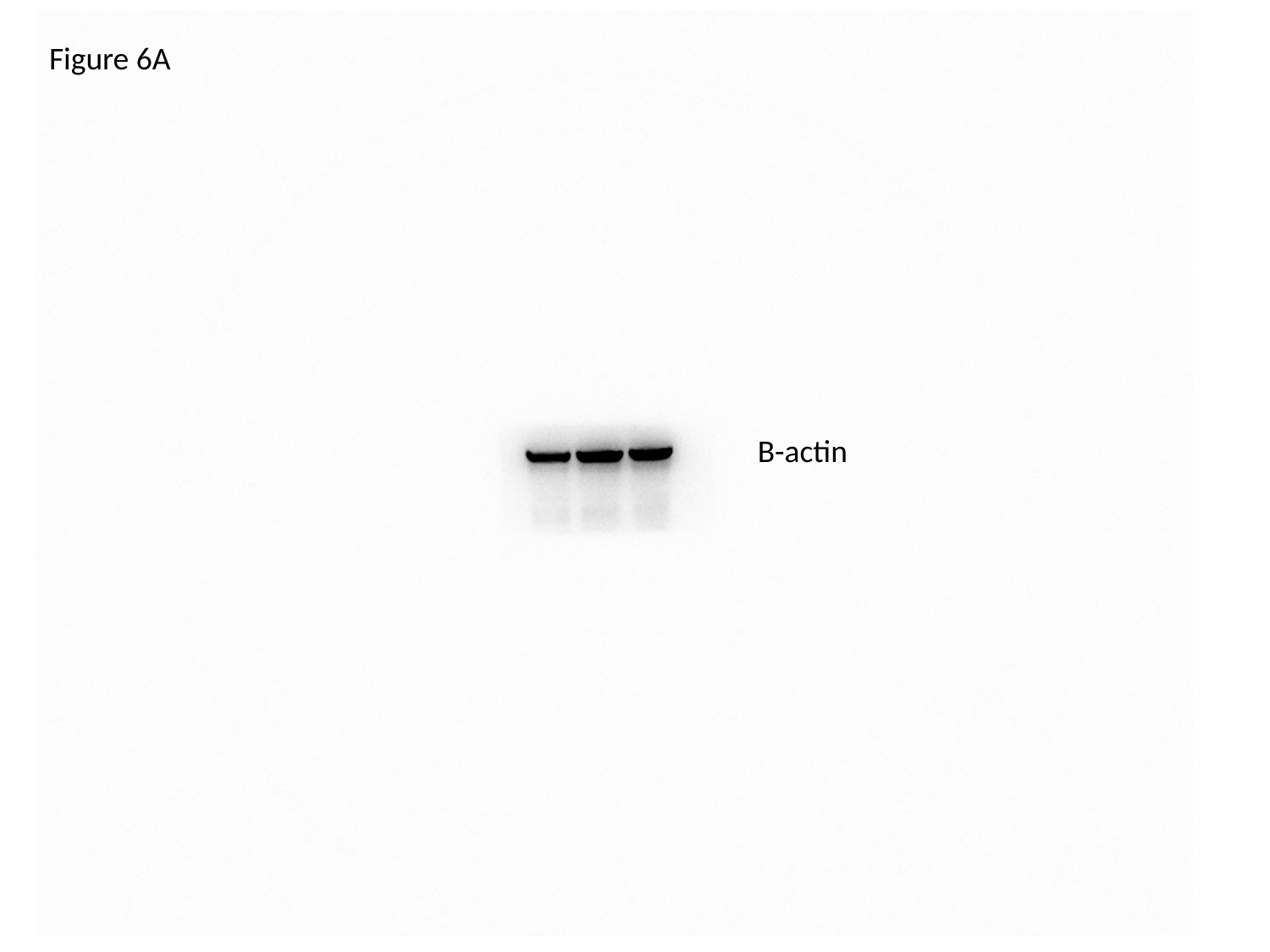

Figure 6A
B-actin

## Slide 7
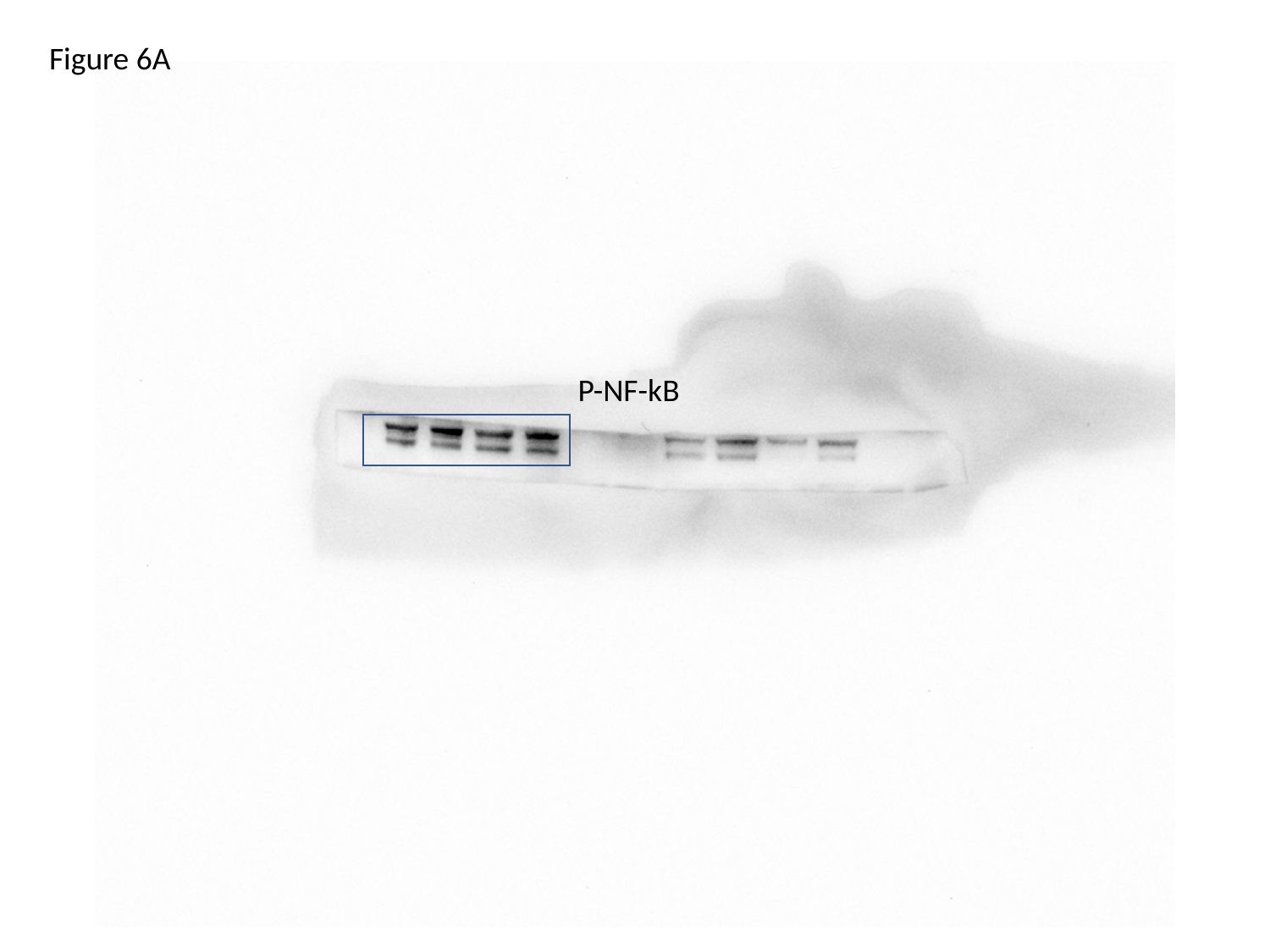

Figure 6A
P-NF-kB

## Slide 8
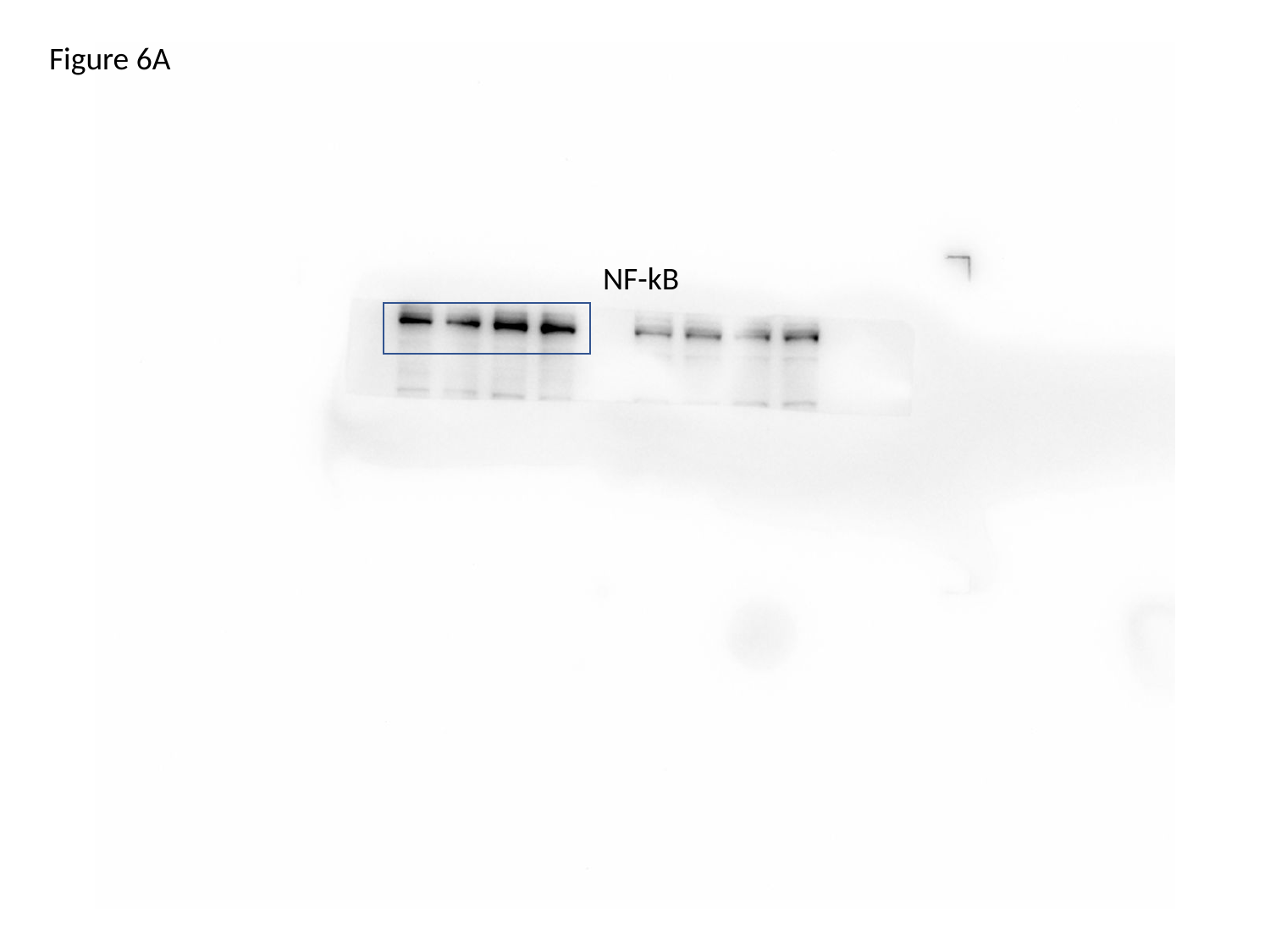

Figure 6A
NF-kB

## Slide 9
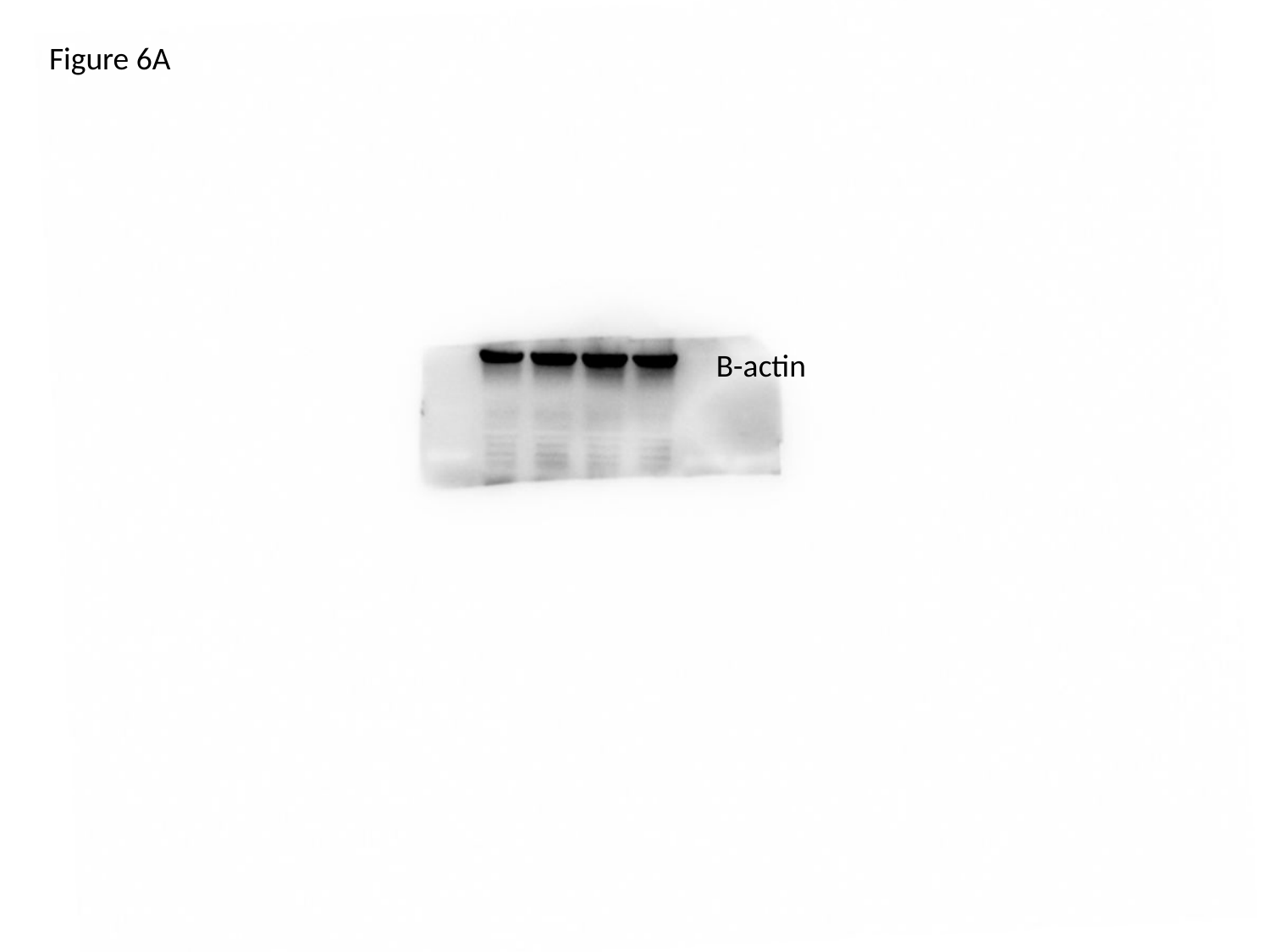

Figure 6A
B-actin

## Slide 10
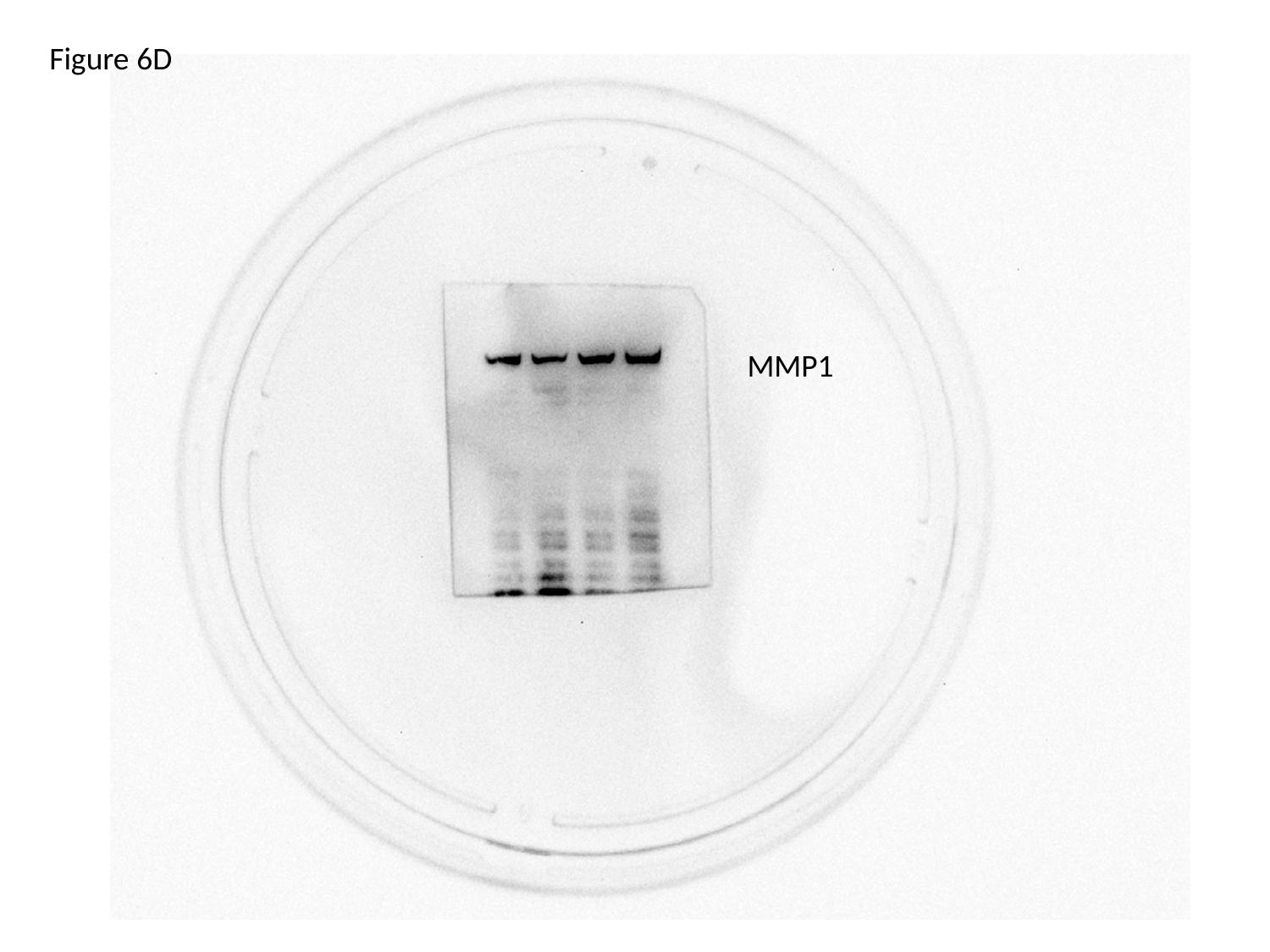

Figure 6D
MMP1

## Slide 11
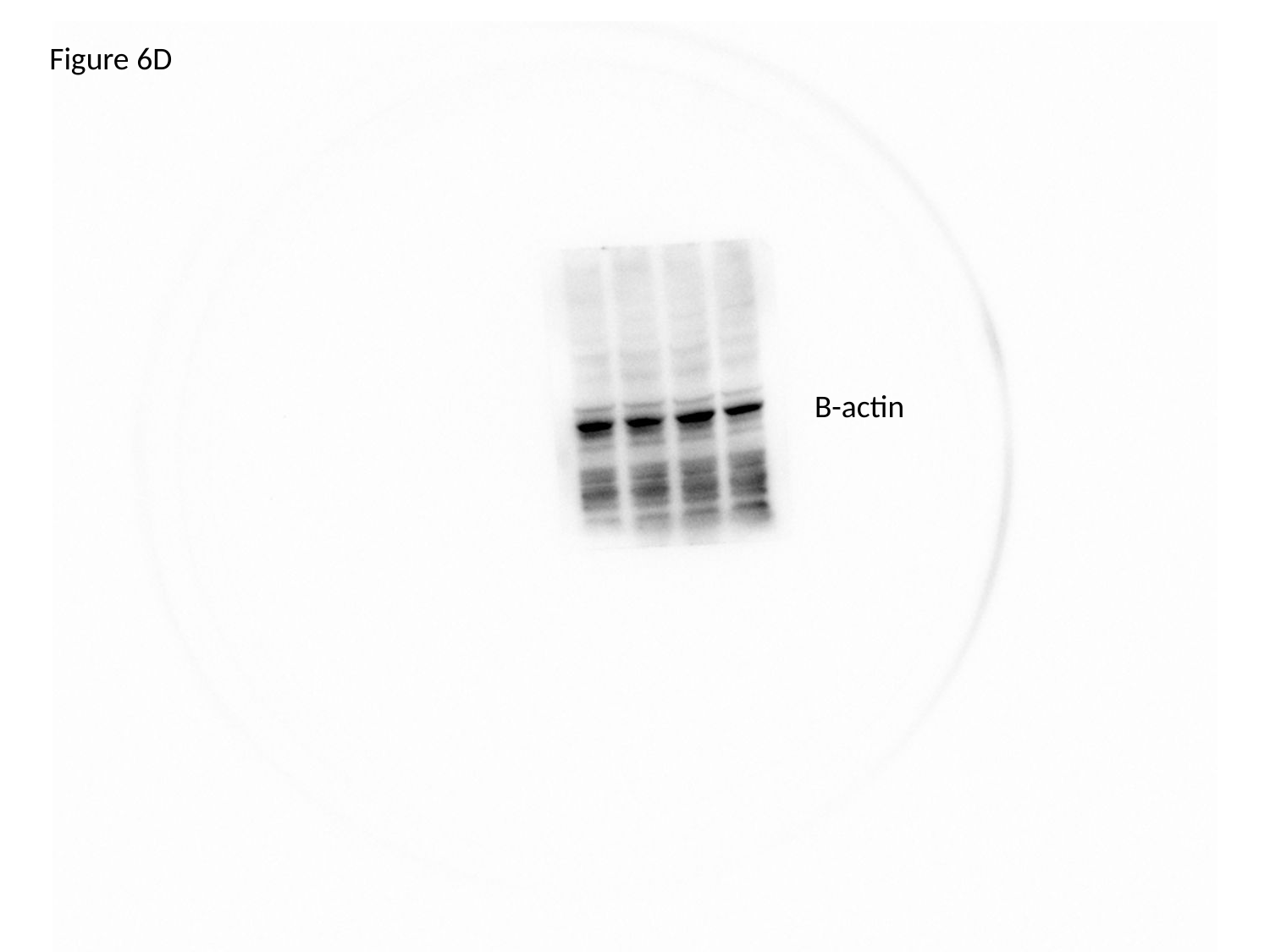

Figure 6D
B-actin

## Slide 12
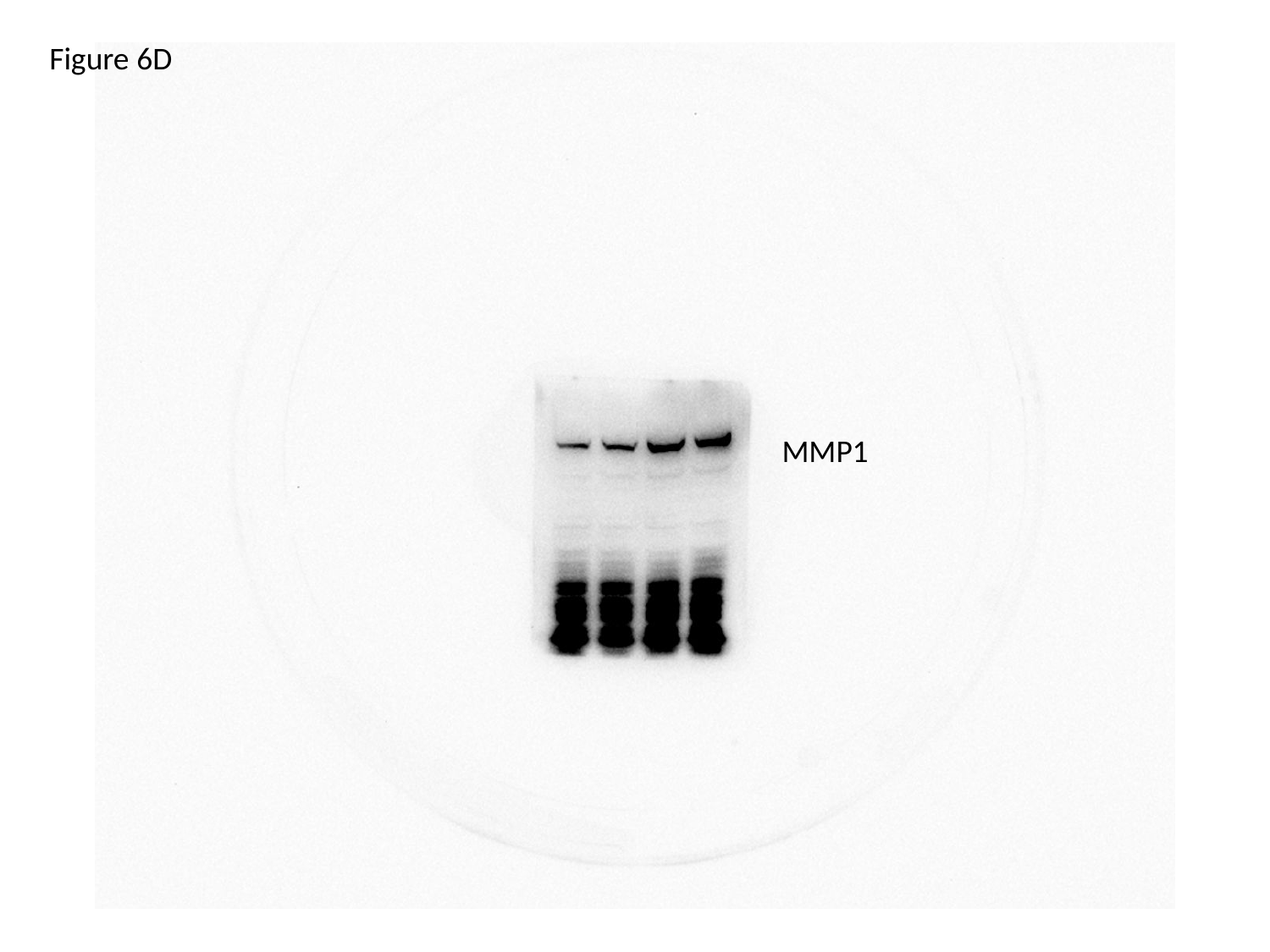

Figure 6D
MMP1

## Slide 13
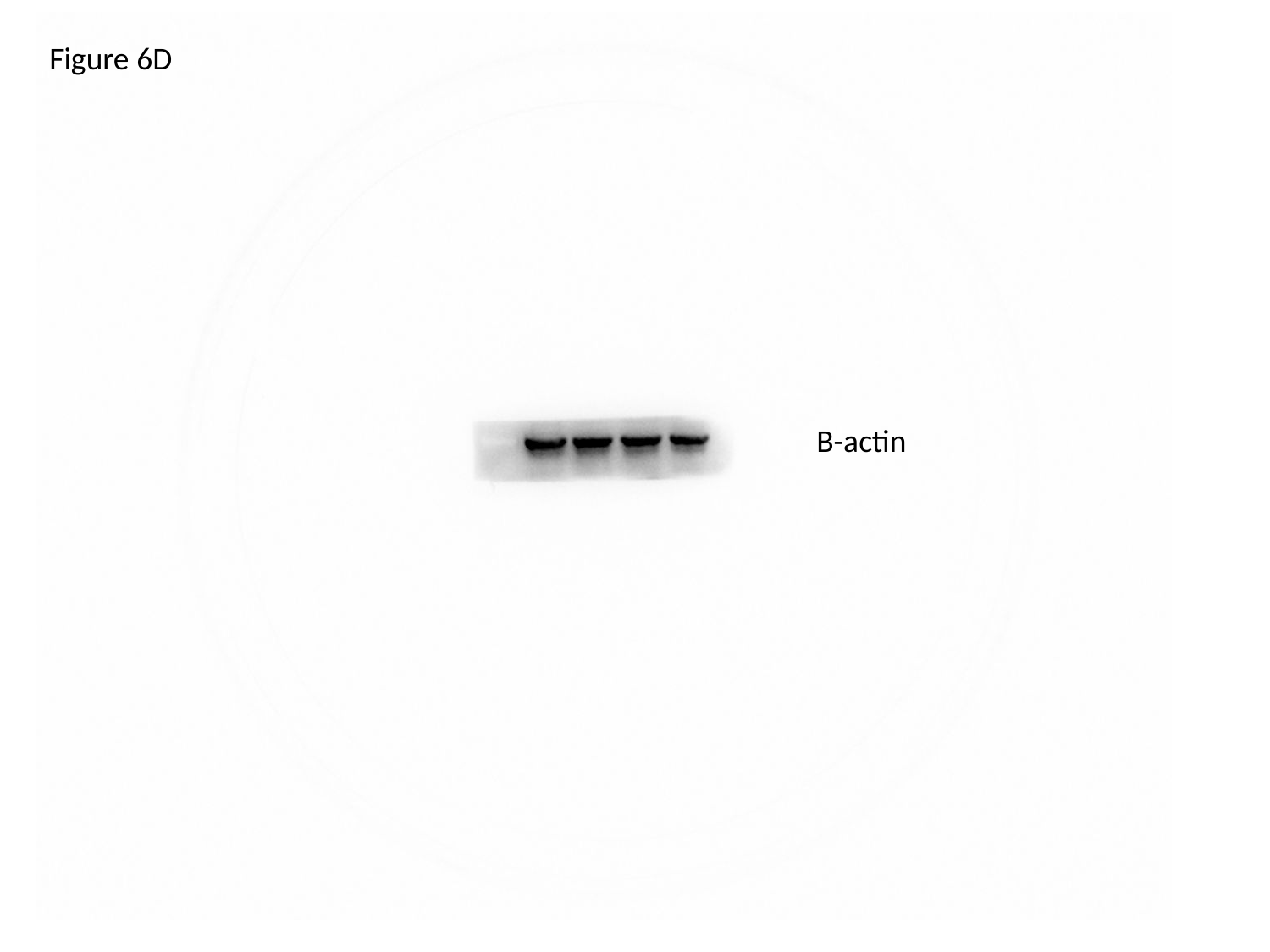

Figure 6D
B-actin
